# Pathogenicity and virulence of West Nile virus revisited eight decades after its first isolation

**DOI:** 10.1080/21505594.2021.1908740

**Published:** 2021-04-12

**Authors:** Juan-Carlos Saiz, Miguel A. Martín-Acebes, Ana B. Blázquez, Estela Escribano-Romero, Teresa Poderoso, Nereida Jiménez de Oya

**Affiliations:** a Department of Biotechnology, National Institute for Agricultural and Food Research and Technology (INIA), Madrid, Spain; b Molecular Virology Group, Department of Experimental and Health Sciences, Universitat Pompeu Fabra, Barcelona, Spain

**Keywords:** West Nile virus, molecular biology, transmission, epidemiology, immune response, clinical presentation, pathogenesis, diagnosis, vaccines, antivirals

## Abstract

West Nile virus (WNV) is a flavivirus which transmission cycle is maintained between mosquitoes and birds, although it occasionally causes sporadic outbreaks in horses and humans that can result in serious diseases and even death. Since its first isolation in Africa in 1937, WNV had been considered a neglected pathogen until its recent spread throughout Europe and the colonization of America, regions where it continues to cause outbreaks with severe neurological consequences in humans and horses. Although our knowledge about the characteristics and consequences of the virus has increased enormously lately, many questions remain to be resolved. Here, we thoroughly update our knowledge of different aspects of the WNV life cycle: virology and molecular classification, host cell interactions, transmission dynamics, host range, epidemiology and surveillance, immune response, clinical presentations, pathogenesis, diagnosis, prophylaxis (antivirals and vaccines), and prevention, and we highlight those aspects that are still unknown and that undoubtedly require further investigation.

## The virus

**Figure 1.**
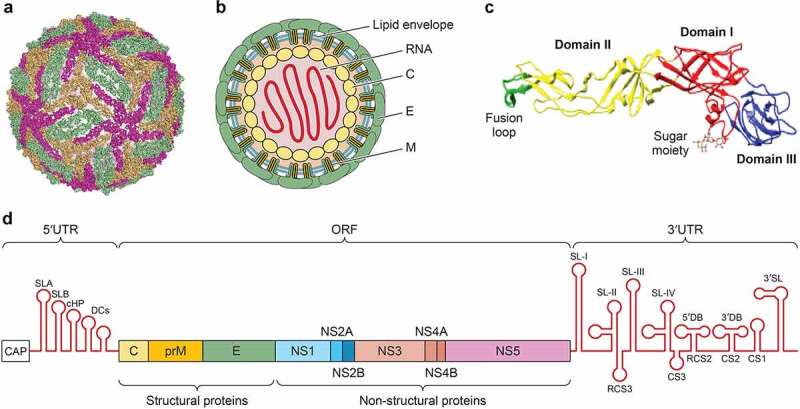
**Structure and genome organization of WNV**. A) Surface representation of WNV particle displaying the arrangement of E glycoprotein (protein data bank accession 3J0B). B) Schematic view of WNV particle. C) Crystal structure of an E glycoprotein monomer (protein data bank accession 2I69). D) WNV genome organization. The mature proteins produced by cleavage of the single ORF are denoted by boxes. See text for details

West Nile virus (WNV), an arbovirus (arthropod-borne virus) transmitted by mosquitoes, is a small (about 50 nm in diameter), spherical, enveloped flavivirus (family *Flaviviridae*) whose genome consists of a single-stranded RNA molecule of positive polarity that encodes three structural and seven non-structural proteins [1]. The genomic RNA is enclosed within a nucleocapsid formed by the capsid (C) protein, which makes up the core of the virion and is enveloped by a lipid bilayer derived from the host cell (Figure 1). Mature virions have a smooth outer surface with no projections or spikes, which is constituted by 180 copies each of the membrane (M) protein and the envelope (E) glycoprotein. These proteins are organized as 90 antiparallel homodimers arranged in three distinct symmetry environments, hence resulting in a particle of icosahedral symmetry [2] that exists as a dynamic and heterogeneous population resulting in a “breathing” virion, which may have relevance for virus-receptor interactions [3].

### Genome

WNV genome is a single-stranded RNA molecule of positive polarity of about 11,000 nucleotides in length that encodes a polyprotein in a single open reading frame (ORF) flanked by two untranslated regions (UTRs) located at the 5ʹ and 3ʹ ends of about 100 and 400–700 nucleotides in length, respectively (Figure 1) [1]. These 5ʹand 3ʹ UTRs fold into RNA secondary structures as stem-loops (SLs), hairpins (HPs), cyclization sequences (CSs), and pseudoknots (PKs) that are conserved among diverse flaviviruses, despite nucleotide divergence, and that interact with each other to act in several steps of the viral life cycle (replication, transcription, and translation) [4].

The genome contains a 5ʹ-cap structure (m(7)GpppAm) necessary for optimal infectivity of WNV RNA. This structure is methylated at the guanine N-7 and the ribose 2ʹ-OH positions of the first transcribed adenine, and it has been related to circumvention of innate immunity by evading certain components of interferon (IFN) response [4]. Consequently, viruses defective in this methylation mechanism can replicate but are attenuated *in vivo* [5]. These RNA modifications have also regulatory activities important for cellular gene expression [6].

The 3ʹ UTR lacks a 3ʹ poly-A tail but ends with a conserved CU_OH_, and is a key determinant of WNV virulence [6,7]. The SLs and PKs of the 3ʹ UTR are critical for the formation of subgenomic RNAs and have a relevant role in the suppression of the host innate immunity and adaptation to different hosts. Mushroom-like structures are essential for replication and translation, and the final section is needed for circularization, RNA synthesis, and replication [6,7].

### Structural and non-structural proteins

The ORF is translated into a single polyprotein that is co- and post-translationally processed by viral and cellular proteases to render ten major viral proteins: three structural (C, prM/M, and E) and seven non-structural (NS1, 2A, 2B, 3, 4A, 4B, and 5) [1].

The C protein is implicated in the nucleocapsid formation by association with the genomic RNA conferring it the chaperoning activity, being crucial for viral assembling and replication [8]. It can be detected in the cytoplasm, nuclei, and nucleolus (through importins) of the cell, and it has been related to the induction of apoptosis [9], and disruption of the blood-brain barrier (BBB), helping virus dissemination [10]. The M is a transmembrane glycosylated protein produced by cleavage of the prM by a furin-like protease in the trans-Golgi to render mature virions [4]. The E is also a transmembrane glycosylated protein involved in receptor binding, viral entry, and membrane fusion, and is the most immunogenic one [2]. E glycosylation is important for efficient transmission and neuroinvasiveness [11]. The E protein has three domains (Figure 1), DI implicated in viral fusion, DII, and DIII, an immunoglobulin-like domain that mediates the homodimerization of the protein, is involved in receptor binding, and contains multiple epitopes that are recognized by neutralizing antibodies (NAbs). Upon acid exposure, the E undergoes conformational rearrangements, exposing the fusion loop of the DII to enable the viral fusion of the virion with cellular endosomal target membranes.

The NS1 glycoprotein can be secreted and is implicated in replication, virulence, immunomodulation, and pathogenesis [12]. Intracellular NS1 localizes to WNV replication sites and is an essential cofactor for viral replication [13], while its cell surface and secreted forms act as immunomodulators [14]. A larger NS1-related protein (tNS1ʹ), produced by a ribosomal frameshift, has been related to neuroinvasiveness [15]. The NS2A is involved in intracellular membrane rearrangements and virion assembly [16], and has an immunomodulatory role, as it inhibits IFN-α/β production [17]. The NS2B is the co-activator of the NS3 viral serine protease [4]. The NS3 encodes a trypsin-like serine protease at its N-terminal, only active when tethered to its NS2B cofactor, which cleaves the viral polyprotein. NS3 also encodes helicase, nucleoside triphosphatase, and RNA triphosphatase activities important for viral replication [4]. The NS4A is involved in membrane rearrangements, in inhibition of IFN signaling [18], in the unfolded protein response [19], and probably acts as a cofactor regulating ATPase activity of the NS3 helicase [20]. The NS4B plays a major role in WNV inhibition of IFN signaling, and it may be implicated in the formation of the viral replication complex [21]. The NS5 colocalizes with dsRNA at the viral replication complex and has two different enzymatic activities. The N-terminal encodes the methyltransferase required for capping of viral RNA, while the C-terminal encodes the viral RNA-dependent RNA polymerase (RdRp) in charge of genome replication, which occurs in association with intracellular membranes of the endoplasmic reticulum (ER) [22], and is also a potent antagonist of IFN signaling [4].

## Virus cell host interactions

### Early steps: Attachment, entry, and fusion

**Figure 2.**
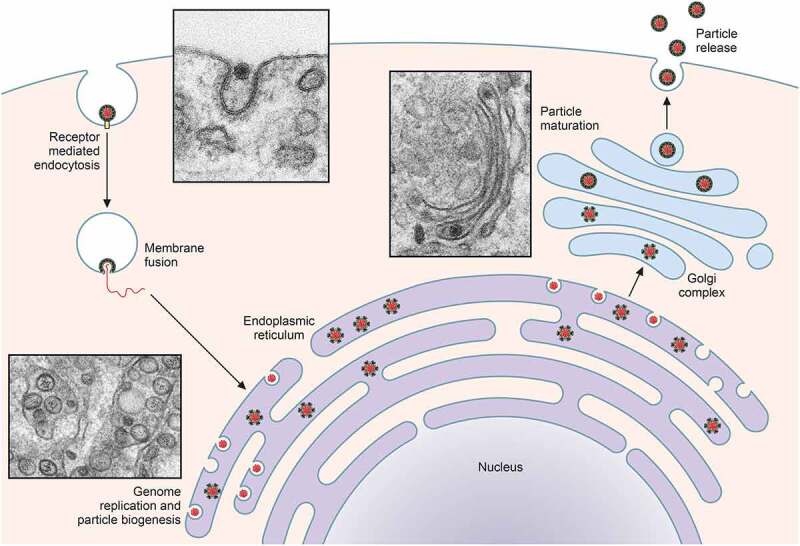
**Schematic view of WNV infectious cycle**. The major steps of WNV infection, including receptor-mediated endocytosis, membrane fusion and genome release, intracellular membrane rearrangements associated with viral replication, immature virion budding into the ER, particle maturation through the secretory pathway, and mature virion release are schematized. See text for details. Electron micrographs to illustrate selected steps of WNV infectious cycle were reproduced under the terms of the creative commons attribution-noncommercial (CC BY-NC 4.0) from [1]

WNV replicates in cells of different origin (insect, mammalian, and avian), and, thus, it uses either conserved or different receptors for viral entry depending on the cell. The infection is initiated by the binding of the virion to its cellular receptor (Figure 2). As detailed below, glycosaminoglycans, c-type lectins like DC-SIGNR, the mosquito mosGCTL-1, TIM phosphatidylserine binding protein, integrin α_v_β_3_, and the ubiquitin ligase CBLL1 have been proposed as cellular receptors for WNV, and proteins from the G-coupled receptor kinase (GRK) family have been reported to act as cofactors that facilitate viral entry and replication.

Viral particles are internalized into host cells via a clathrin-dependent mechanism and later transported to endosomal compartments with the involvement of cellular actin and microtubules, and the action of the small GTPase Rab5 (Figure 2) [23]. Inside the endosome, acidic pH triggers rapid conformational changes on the E protein that result in its fusion with the endosomal membrane, thus allowing the release of the nucleocapsid into the cytoplasm for genome uncoating [24]. As the pH of the endosome acidifies, viral fusion occurs in the presence of cholesterol on the target membrane [25].

### Translation and replication complex assembly

Viral RNA recruits the 40S ribosomal subunit and other associated cellular components to reach the AUG start codon to initiate translation, giving rise to a single polyprotein that, after being processed in the cytoplasm, produces the structural and NS proteins necessary for viral replication and virion assembly.

RNA replication requires the synthesis of a minus-strand RNA that acts as a template for new positive-strand viral genomes [4]. Infected cells undergo notable intracellular membranes reorganizations of the ER that lead to different well-defined structures to establish the viral replication complex. Viral replication takes place at vesicle packets (VPs), which are invaginations of the ER membranes in contact with the cell cytoplasm through pores and contain dsRNA replication intermediates [26]. Once virions are assembled, they bud into the ER (Figure 2), where both cholesterol and fatty acids help the WNV-induced membrane rearrangements [22,27]. Proteasome activity has also been described as important for viral replication [28] and to interfere with the IFN signaling machinery, probably influencing the evasion of the innate immune response [29]. Replication of viral RNA and accumulation of NS proteins at the ER induce its stress, activates the unfolded protein response [30,31], and promotes apoptosis of the infected cells [32].

### Particle maturation and viral egress

Immature viral particles, assembled at the ER, traffic through the secretory pathway and complete its maturation by the proteolytic processing of the prM by a furin-like protease, giving rise to the M protein inside the trans-Golgi network to render mature viral particles that egress from the infected cells (Figure 2) [1].

## Molecular classification

**Figure 3.**
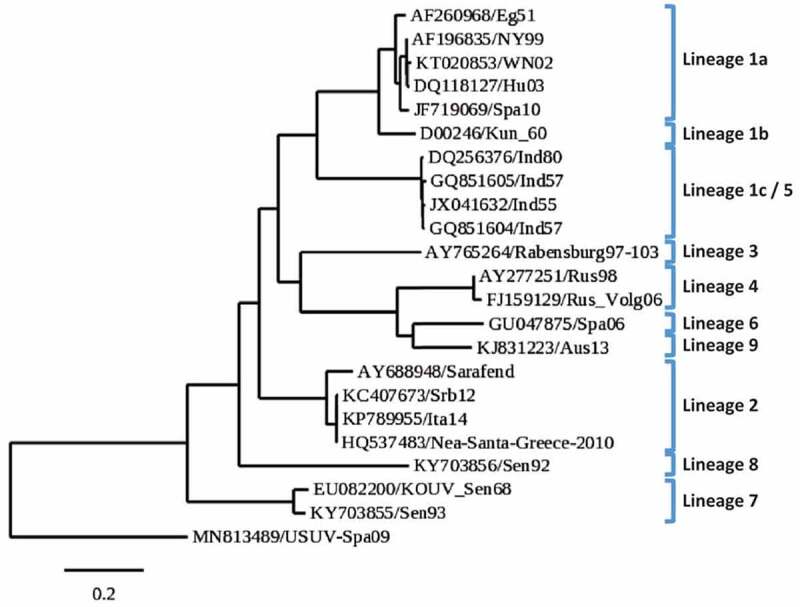
**Phylogenetic analysis based on complete genome nucleotide sequences of the different West Nile virus lineages**. GenBank accession numbers, geographic origin and year of isolation of samples are shown. The scale bar depicts genetic distance. The Usutu virus USUV-Spa09 strain was used as out-root

WNV is classified into several lineages (Lin) [33] that do not consistently correlate with its geographical distribution (Figure 3), although only Lin 1 and 2 have been involved in human outbreaks of WN encephalitis, and both are now endemic in Europe (https://www.ecdc.europa.eu/en/west-nile-fever/facts/factsheet-about-west-nile-fever). Lin 1 is the most widely distributed and is sub-classified into three clades (1a, 1b, 1c). Clade 1a is globally spread, clade 1b, or Kunjin virus (KUNV), mainly circulates in Australia, and clade 1c (now reassigned to Lin 5) has only been described in India [34]. Lin 2 has been reported in Africa, Europe, and the Middle East [35]. Lin 3, or Rabensburg virus, Lin 4, and a putative Lin 6 (closely related to Lin 4) were described in Czech Republic, Russia, and Spain, respectively [33]. Lin 7, initially classified as Koutango virus [36], and Lin 8 were identified in Senegal [37]. Finally, a Lin 9, proposed to be a subclade of Lin 4, was isolated in Austria [38].

## Transmission cycle

WNV is maintained in nature in an enzootic cycle between invertebrates (mosquitoes) and vertebrates, mainly birds, although equids and humans are accidental hosts.

### Vectors

Mosquitoes are the natural vectors of WNV and their ecobiology (distribution, feeding activity, and capacity) is a key issue in virus behavior. WNV has been detected in over 150 species of mosquitoes belonging to, at least, 11 genera. However, its main vectors are those of the *Culex pipiens* L. complex [39]. This complex is convoluted and comprises many different members, such as *Cx. pipiens, Cx. quinquefasciatus, Cx. australicus*, and *Cx. Globocoxitus*, the first two being the most relevant vectors of WNV. Both species are distributed in Africa, Asia and the Americas [39], and *Cx. pipiens* and *Cx. quinquefasciatus* are also present in Europe [40] and Australia [41], respectively. *Cx. pipiens* is distributed in temperate regions, while *Cx. quinquefasciatus* habits in both tropical and subtropical areas [42], although they also share niches, where hybridization occurs between them [39]. *Cx. quinquefasciatus* is considered to be opportunistic in both mammals and birds in terms of host feeding preferences [42]. *Cx. pipiens* has two recognized forms, i.e., *Cx. p. pipiens* and *Cx. p. molestus*, which differ in their physiology and behavior [43]. Both forms are synanthropic, although *Cx. p. molestus* is more frequently found in human habitats [43] due to its mamophilic behavior, pointing to it as a key player of the zoonotic transmission of WNV to humans, while *Cx. p. pipiens* plays a more significant role in the natural ecology of WNV because it is mainly ornithophilic, suggesting that it is relevant for the maintenance of WNV in its natural enzootic cycle.

Other species belonging to the genus *Culex* are also considered as relevant for the global transmission dynamics of WNV. These species include preferentially ornithophilic mosquitoes such as *Cx. annulirostris* distributed in Australia and Asia, *Cx. modestus* present in Europe, Asia, and Africa, *Cx. perexiguus* circulating in Africa and Europe and that also feeds on humans, and *Cx. restuans* present in the Americas and that has a lower preference for feeding on mammals. Moreover, there are also other species with both mamophilic and ornithophilic behaviors, such as *Cx. antennatus* in Africa, which is also anthropophilic, and *Cx. tarsalis*, one of the main vectors in the US and Mexico, or *Cx. salinarius* in the US [44]. Other species of mosquitoes that have also been implicated in the epidemiology of WNV include some distributed in Asia, such as *Cx. pseudovishnui* and *Cx. vishnui*, both with a preferential feeding in cattle and pigs, although the latter also feeds on humans and chickens, and *Cx. tritaeniorhynchus* that preferentially feeds on cattle and pigs and is distributed in Australia, Asia, and Africa.

Apart from species of the genus *Culex*, which is considered the main one implicated in both enzootic and zoonotic WNV transmission cycles worldwide, other species are considered secondary vectors, such as *Culiseta melanura* (Coquillett) in the US, and mosquitoes of the genus *Aedes*, such as *A. albopictus* [1], distributed on all continents except Antarctica [45] and *A. vexans* [1], distributed throughout the Holarctic region [46]. Likewise, species belonging to the genus *Ochlerotatus*, like *O. japonicus* and *O. triseriatus* [1], or some of the genus *Anopheles*. These species, although less important for the WNV cycle, can serve as a bridge for the transmission of the virus to humans due to their biting behavior. Besides, the virus has also been detected in other mosquito genera, such as *Mansonia* [47], *Coquillettidia, Aedeomya, Mimomyia, Psorophora, Deinocerites*, and *Uranotaenia* [48].

The virus has also been isolated in other arthropods, like hard (*Amblyomma, Argas, Dermacentor, Hyalomma, Rhipicephalus*) and soft ticks (*Ornithodoros*) species, but their role in WNV activity in nature is unclear.

Virus maintenance is the result of complex interactions of different parameters and events at a given time and place, as the coexistence of suitable vectors and hosts and virus fitness. The vectorial capacity can be measure as the capacity of a vector population to transmit the virus to a susceptible host. It is a complex parameter that encompasses vector population density with respect to the host, transmissibility (effectiveness to transmit the virus when feeding), rate of feeding/day in the host, and daily half-life of the mosquito, among others [49]. Vector competence is understood as the ability to acquire and transmit the pathogen, and hence, is a pivotal component of the overall vectorial capacity of a given population of mosquitoes. Vector competence has been extensively studied for WNV and other arboviruses, although there is variability on the results due to the lack, in many cases, of uniformity in the methods used [50]. Importantly, and although is a crucial requirement, a vector with a high competence will play a low role in the viral cycle if adequate behavioral characteristics, such as preferential feeding in the proper host, are not occurring. In any case, vector competence is intimately related to viral evolution; therefore, genetic changes occurring in viral populations can alter vector competence

### Viral diversity

Similarly to what occur with other RNA viruses, WNV behaviors as a quasispecies due to their genetic diversity [51] that is both a source and a consequence of host/vector adaptation. WNV replicates in very phylogenetically distant different hosts and vectors species, in which viral populations have different replicative fitness that is sensitive to genetic changes. Hence, viral diversity can drive to the emergence of new strains. In this sense, it was reported that E glycosylation was related to efficient virus transmission by *Cx*. vectors [11]. However, more recent studies have shown that the competence of *Cx*. vectors is not affected by N-linked glycosylation of the E protein, but by amino acid polymorphisms, which also affect avian competence [52]. In fact, strain WN02, characterized by a single amino acid change in protein E, which displaced strain NY99 after its initial outbreak in the US, is transmitted more efficiently by species of the genus *Cx* [53]. This strain currently co-circulates with a later-onset strain, SW03, characterized by unique amino acid changes in the NS4 and NS5 proteins. Experimentally, both strains appear to have a similar replicative capacity in mosquitoes of the genus *Cx* [54], although SW03 is spreading more rapidly in the southwestern US [55]. Similarly, a single mutation in the NS3 protein has been associated with a possible increase in viremia and also with increased virulence in American crows [56].

The impact of the vertebrate and invertebrate host with regard to viral diversification and to the gain/loss of fitness in viral replication has also been addressed. A high diversity of WNV competing genomes leads to increase fitness in vector mosquitoes, but not in avian hosts [57]. A greater intra-host viral diversification occurs in mosquitoes [58], in which existence [59] and lack [60] of bottleneck phenomena that may alter the genetic diversity of WNV genomes have been reported. Furthermore, it has been reported that a genetic drift occurs inside the mosquito vector, but that such reduction of genetic diversity is recovered by tissue-associated viral population expansions and that the intra-host viral selection is differently affected by the different mosquito species. Interestingly, the population arising from this invertebrates vectors loss fitness on vertebrate hosts [61]. Viral diversification within mosquitoes seems to occur preferentially in regions that are targeted by the vector immune innate system, that mainly rely on RNAi [62] that may shape purifying selection. Therefore, in the saliva of the mosquito highly diverse, but unique populations are found. However, this diversity is reduced after host-switching, suggesting that a stronger purifying selection occurs in birds [58]. The complex interaction of innate immune mechanisms in the vertebrate host may also be behind the damped viral diversification. All these complex intra- and inter-host interactions lead to the low evolutionary rate found in mosquito-borne viruses in comparison to other RNA viruses.

On the other hand, persistently infected hosts and vector vertical transmission seem to also play a role in the virus cycle in regions where vectors are not annually active [63], but their role in virus maintenance during overwintering periods in temperate regions remains to be confirmed.

### Birds

More than 300 species of birds, belonging to more than 20 different families, have been implicated in the WNV cycle [64], being those of the Passeriformes order, mainly of the family Corvidae, the most important players, as they are highly efficient virus amplifiers and, thus, competent-hosts to keep the viral transmission in nature. Indeed they develop viremias with titers compatible with the reported threshold (10^4^–10^5^ pfu/ml) needed for efficient transmission to the vectors by blood-feeding [64]. As detailed below, WNV can produce disease and death in several bird species, and bird-to-bird transmission has been reported, in nature and experimentally, by ingestion and by close contact, most probably due to the high viral loads detected in oral and cloacal cavities and the pulp of growing feathers [64–66]

### Human, horses, and other mammals

Horses and humans are considered “dead-end” accidental hosts because WNV-related vectors are mainly ornithophilic and because the viremias reached in them are often inadequate to maintain the virus cycle. However, since morbidity and mortality are observed in both species, these infections have a great economic and human health repercussion [67]. In addition to mosquito bites, sporadic transmission has also been documented by blood transfusions and transplants in humans, and by transplacental and lactation routes in humans and experimentally infected animals [68–71].

Several other vertebrates are also naturally exposed to WNV, including wild fauna (squirrels, chipmunks, house mice, hamsters, bats, bears, wolves, tigers, lions, civets, striped skunks, raccoons, and crocodiles) and other mammalians in close contact with humans (dogs, cats, sheep, pigs, and cows, among others), but their role in maintaining WNV cycle in nature is still uncertain [72].

## Epidemiology and surveillance

### Europe and the Mediterranean basin

WNV-specific antibodies (Abs) in humans were documented for the first time in 1958 in Albania, and the first outbreaks in humans and horses were reported in 1962–1963 in France. Nevertheless, and although the virus was isolated from mosquitoes, ticks, and birds, and human seropositivity was sporadically reported in several countries, WNV was not considered a public health concern until 1996, when a large outbreak in humans occurred in Bucharest (Romania) with over 390 confirmed cases (European Center for Disease Prevention and Control, “Historical data, http://ecdc.europa.eu/en/healthtopics/west_nile_fever/West-Nile-fever-maps/Pages/historical-data.aspx). Since then, cases were reported around the continent in animals and humans due to Lin 1 strains until 2004, when Lin 2 was first isolated in Europe from a goshawk in Hungary [73] that later spread through the continent causing several outbreaks in humans and horses, being now both Lin endemic in the region. The most recent data (as of 8 October 2020) shows an accumulated number of human cases of 285 and 31 deaths reported to ECDC since the start of the 2020 transmission season (https://www.ecdc.europa.eu/en/publications-data/west-nile-virus-europe-2020-human-cases-compared-previous-seasons-updated-8).

At the EU level, WNV infection is notifiable for humans and equids through The European Surveillance System (TESSy) of the European Center for Disease Prevention and Control (ECDC) (http://ecdc.europa.eu/en/activities/surveillance/TESSy/Pages/TESSy.aspx) that publishes weekly WNV epidemiological and geographical distribution updates. Equine and bird cases are defined according to the Terrestrial Animal Health Code (https://www.oie.int/index.php?id=169&L=0&htmfile=chapitre_wnf.htm) of the World Organization for Animal Health (OIE), and are collected through the Animal Disease Notification System (ADNS) (https://ec.europa.eu/food/animals/animal-diseases/not-system_en). While the report of WNV infections among birds is voluntary, the report of equine encephalomyelitis is mandatory, but its surveillance is mostly passive and only a few countries have active programs.

In the Mediterranean basin, the first WNV case was reported in the 1950s in a febrile child in Egypt [74], where the virus is now endemic. At that time the virus was isolated also from a febrile child in Israel, where outbreaks occurred in the following years, although retrospective analyses suggested that the virus was circulating there from the 1940s [75]. Since then, no infections had been documented until 2000, when an outbreak with 417 serologically confirmed cases and 35 deaths was described; from then until now outbreaks are reported every year in the region (European Center for Disease Prevention and Control, “Historical data”, https://www.ecdc.europa.eu/en/west-nile-fever/surveillance-and-disease-data/historical). WNV circulation has also been documented in humans and/or birds, horses, and dogs in Algeria, Morocco, Tunisia, and Turkey [76,77] but, despite recent efforts to harmonize surveillance programs across the region [78], so far, none has yet been implemented.

### The Americas

WNV emerged in the Western Hemisphere in the US in the summer of 1999, when a high mortality among birds and an unknown sickness of horses preceded human cases that accounted for 62 deaths [79]. Although the introduction way is still unknown, the initially isolated strain (NY99) was closely related to a 1998 goose isolate from Israel [79] and spread quickly across the country until it was replaced by WN02 strain in 2002, which is now dominant there [80]. Nowadays WNV counts for up to 2,330 fatalities, over 24,700 cases of neuroinvasive disease, and more than 50,000 diagnosed human infections, with remarkable peaks in 2002, 2003, and 2012 (http://www.cdc.gov). In addition, until the massive vaccination of horses, more than 25,000 accumulated cases had been documented.

WNV disease is a notifiable condition in the US (https://www.cdc.gov/westnile/resourcepages/survResources.html) and is reported to the CDC (https://wwwn.cdc.gov/nndss/conditions/arboviral-diseases-neuroinvasive-and-non-neuroinvasive/case-definition/2015/) through a national arboviral surveillance system (ArboNET), which, in addition to human disease, maintains data on infections among blood donors with suspected viremia, veterinary cases, mosquitoes, dead birds, and sentinel animals. However, being a passive system, surveillance data for the non-neuroinvasive disease should be interpreted with caution.

Outside the US, the first human infection in the continent was confirmed in Canada in 2002, where a peak of 2,215 cases was reported in 2007, being now endemic there [81]. Canada conducts humans, horses, birds, and mosquitoes surveillance throughout the country (https://www.canada.ca/en/public-health/services/diseases/west-nile-virus/surveillance-west-nile-virus.html).

In Mexico, the virus was detected in horses in 2002 and in humans in 2004. After that, WNV spread to El Salvador, Belize, Guatemala, Costa Rica, and Nicaragua, where a single human case was documented in 2006. WNV activity in the Caribbean was first documented in the Cayman Islands in 2001, and later on, seropositivity has been recorded in birds and/or horses, as well as sporadic human cases, in Jamaica, the Dominican Republic, Guadalupe, Trinidad, Puerto Rico, Haiti, and Cuba [82]. Similarly, the virus was detected in South America in horses in Colombia in 2004, and, from there on seropositivity and/or sporadic cases have been described in birds and horses in Venezuela, Brazil, Bolivia, and Argentina, where four encephalitis human cases were reported [83]. However, no large human outbreaks have been detected in these regions (https://www.paho.org/hq/index.php?option=com_topics&view=rdmore&cid=2195&Itemid=40782&lang=en).

The low impact of WNV in countries south of the US evidences high contrasts between both regions. Several explanations have been suggested to support it, as misdiagnosis due to the circulation of other flaviviruses (cross-reactions), protection resulting from previous exposure to flaviviruses, suboptimal disease surveillance [84], or even the circulation of attenuated strains [85]. However, only Mexico (http://www.dof.gob.mx/nota_detalle.php?codigo=5389045&fecha=16/04/2015) and Brazil (https://www.paho.org/pt/brasil) have active surveillance programs.

### Africa

Even though WNV was first isolated in Uganda [86], little information is available about its impact in Africa. To date, only mild disease and no human deaths have been documented. Human seropositivity has been reported in Uganda, Sudan, the Democratic Republic of the Congo, Kenya, Nigeria, Sudan, Senegal, Mali, Madagascar, and South Africa, where avian, monkey, and domestic animal samples were also positive [87]. In South Africa, WNV infections are also usually mild, although human epidemics were reported in 1974 and 1984 [88]. This relatively low incidence has been attributed to the same factors mentioned above for the Americas.

### The Middle East, Asia, and Oceania

The epidemiological scenario in these regions and its potential impact on health has been poorly addressed. Seropositivity and sporadic detection of WNV-RNA have been reported in humans in Djibouti, Egypt, Iran, Iraq, Jordan, and Lebanon with varied prevalence, but, with few exceptions, no illness has been reported [78]. Likewise, positive horses, dogs, and birds have also been documented in a few studies, and the virus has been isolated from mosquitoes [78]. However, no surveillance programs are implemented in these regions.

In the southern area of European Russia, western Siberia, and adjacent republics of the former Soviet Union (Tajikistan, Kazakhstan, Ukraine, and Turkmenistan) WNV circulation is known since 1963, and strains from different lineages have been isolated from ticks, birds, and mosquitoes, but little evidence of human disease was provided until 1999 [89] when a large outbreak of severe neurological disease involving 318 cases and 40 deaths was reported in Volgograd [90].

In India, WNV has been isolated from mosquitoes and humans, and variable seropositivity has been reported in the country [91], where some cases of acute encephalitis [92], and a few WNV-confirmed pediatric fatalities have occurred [93]. Apart from India, WNV seropositivity has also been described in Pakistan, Myanmar, Thailand, and the Philippines, and Lin 1 and 2 strains have been isolated in Nepal, Indonesia, Malaysia, and Cambodia [94], but again, surveillance studies are scarce.

Similarly, little information is available from China. Positive seroprevalence has been described in horses and birds [95], and WN fever, viral meningitis, and encephalitis have been documented in humans [96], but no data about current surveillance programs are available.

The Australian strain of WNV (KUNV) has been continuously circulating since 1960 with low incidence, resulting in infrequent small outbreaks in humans and horses without fatalities [97]. In 2011, hundreds of horses with WNV-associated neurological disease were observed in New South Wales [98] but, surprisingly, a follow-up survey indicated a very low seroprevalence [99].

WNV/KUNV infections are notifiable in the country, its activity is assessed through sentinel chickens and mosquitoes surveillance programs (https://www.health.wa.gov.au/Articles/A_E/Arbovirus-surveillance-program https://www.health.nsw.gov.au/environment/pests/vector/Pages/surveillance.aspx), and surveillance and monitoring reports are weekly published (https://www.health.nsw.gov.au/environment/pests/vector/Pages/nswasp-weekly-report-2019-20.aspx).

## Immune response

### The role of mosquito saliva

When infected mosquitoes are feeding, WNV is delivered into the host’s dermis and epidermis via saliva, which contains factors that can enhance virus infection [100]. The skin, one of the first barriers of the immune system, is populated by resident innate cells, like antigen-presenting cells (APCs) as Langerhans cells (LCs), and less specialized ones, like keratinocytes with key roles during WNV infection. Circulating and migratory innate cells are attracted to the target site following chemokine gradients released at the inoculation site by cells stimulated through their pattern recognition receptors (PRRs) by both damage- and pathogen-associated molecular patterns [101].

Neutrophils arrive early to the target site, recruited by mast cell degranulation, enhancing early infection by supporting WNV replication. Paradoxically, at late stages, they help control it, as shown in mice which their depletion increases susceptibility [102]. Saliva factors of different mosquitoes have been described to both promote [103] and inhibit mast cell degranulation [104] and also to interfere with later T-cell recruitment [100]. Therefore, these factors can enhance viral infection by diminishing antiviral responses, and also by increasing the infiltration of WNV-target cells [105] with the consequent viral dissemination to secondary lymphoid organs (SLO), thus, helping the establishment of a systemic infection [106]. However, infection outcome is also affected by the diverse effects that mosquito saliva exerts in the immune response [107] and by prior exposition to mosquito bites [105]. So that, saliva alters local hemostasis by augmenting vasodilatation and blood vessel permeability, which increases both immune cell recruitment and viral particle dissemination, and has an immunomodulatory effect mainly due to its influence in cytokine production, with the consequent cell type activation/migration, which shapes the immune responses generated.

### Immune cell targets and receptors

Primary target cells for WNV replication are keratinocytes, dermal dendritic cells (DCs), and LCs, but fibroblasts are also permissive [108]. As mentioned above, several receptors have been implicated in the permissiveness of these cells to the viral adhesion/entry, which are differentially expressed on innate and adaptive immune cells and in different locations of the body and, therefore, have different implications on WNV pathogenesis.

C-type lectin receptors (CLRs) are PRRs that trigger different cellular pathways resulting in pleiotropic outcomes. DC-SIGN and DC-SIGNR, expressed in several immune cells, promote viral infection by interacting with N-linked glycans motives on the viral E protein [109]. Phosphatidylserine (PtdSer)-receptors, such as those belonging to the TIM and TAM family, are also expressed on several innate and adaptive immune cells, and are involved in phagocytosis of apoptotic cells and in the inhibition of inflammatory responses. WNV exploits these receptors to increase viral internalization by a mechanism called “apoptotic mimicry” through the PtdSer and phosphatidylethanolamine that carries and serves as an “eat-me” signal to trigger phagocytosis, thus, increasing virion internalization [110]. Accordingly, TIM-1 and TIM-4 and, to a lesser extent, TIM-3 receptors have been related to increased viral replication [111,112]. Regarding TAM receptors (Tyro3, Axl, and Mertk), their role in WNV infection is somehow complex. A protective role has been suggested in mice for Axl and Mertk by their potential in regulating BBB integrity [113], and also by the capacity of Axl to avoid exacerbated IFN responses that result in the blockage of DCs maturation, reduction of IL-1β, and impairment of T-cell priming [114]. However, a pathogenic role has also been described, since an increased DC-Axl expression seems to impair IFN-I responses in the elderly [115]. In addition, Axl and Tyro 3 increase *in vitro* viral uptake [112], and TAM activation suppresses Toll-like receptor (TLR) signaling, impairing IFN responses and antiviral activity [116,117].

### Innate control of the virus

The innate immune system is the first responder to a pathogen invasion, recognizing common pathogenic features in a nonspecific way that is importantly based on PRRs expression by innate cells which triggers the production of pro-inflammatory cytokines, of which IFNs display essential antiviral functions in the control of WNV infection.

#### Interferons

The protective role of IFN-I (IFN-α and IFN-β) against WNV has been described in transgenic mouse models knocked down for its receptor (IFNAR), in which an enhanced virulence is observed, which is related to lack of viral tropism restriction, an increment of viral burden, and central nervous system (CNS)-related pathology [118]. Type II interferon (IFN-γ) is produced by both innate and adaptive immune cells and signals through the IFNGR receptor complex. WNV infected mice lacking both IFN-γ and IFNGR receptors show higher mortality rates, increased viremia and viral burdens in SLO, early CNS viral entry, and impaired IFN-γ-mediated normal function of γδT cells and DCs [119].

#### Pattern recognition receptors (PRRs)

**Figure 4.**
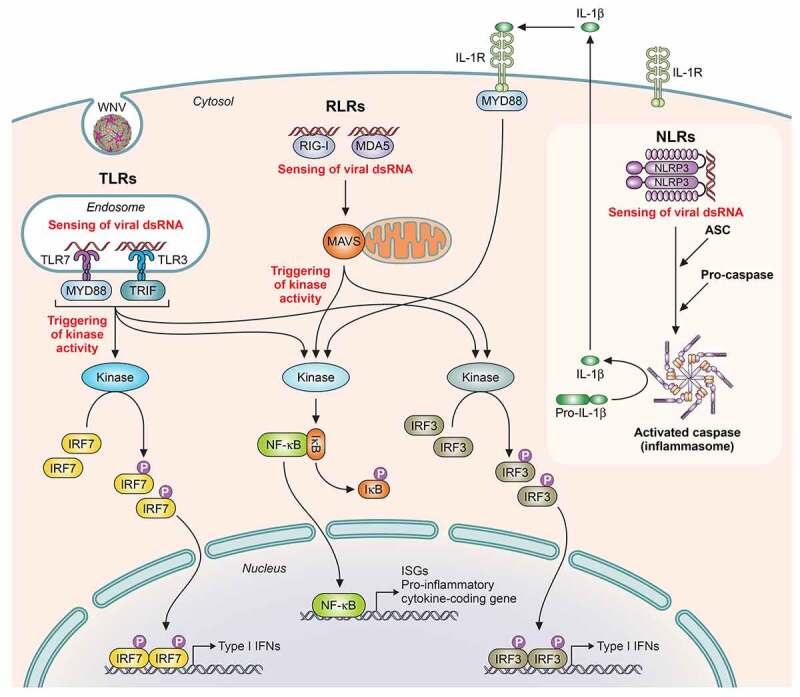
**Schematic representation of pathogen recognition receptors (PRRs) activation implicated in WNV infection**. Upon binding of viral components, PRRs activation induces a downstream signaling cascade with adaptor proteins implicated and catalytic enzyme activities that promotes the activity of transcription factors, each of which capable of inducing the expression of different target genes, such as Type I IFN genes, ISGs (IFN-stimulated genes) and pro-inflammatory cytokine coding genes. PRRs: TLRs (Toll-like receptors, like TLR-7 and TLR-3), RLR (RIG-I-like receptors, like RIG-I [retinoic acid-inducible gene I protein], and MDA5 [melanoma differentiation antigen 5]) and NLR (NOD-like receptors, like NLRP3). Adaptor proteins: MYD88 (myeloid differentiation 88), TRIF (TIR domain-containing adaptor inducing IFN-β), MAVS (mitochondrial antiviral signaling), and ASC (apoptosis-associated speck-like protein, containing a caspase recruitment domain). Transcription factors: IRF (interferon regulatory factors, like IRF7 and IRF3) and NF-κB (nuclear factor kappa B). Adapted from [265]

Among the described PRRs sensing WNV (Figure 4) are TLRs, like TLR-3 and TLR-7, RIG-I-like receptors (RLRs), as RIG-I (retinoic acid-inducible gene I), and MDA5 (melanoma differentiation-associated gene 5), and also NOD-like receptors (NLRs). Its activation leads to common key activities for virus control, as the synthesis of antiviral proteins that impact viral replication, and the secretion of pro-inflammatory cytokines, which act by either amplifying cell-intrinsic antiviral responses or by shaping both innate and adaptive immune responses [101].

Upon WNV infection, mice lacking different genes related to PRRs signaling have shown a higher susceptibility. Thus, IPS-1^−/-^ (IFN-β promotor stimulator-1) mice show higher viral replication and early arrival to the CNS with exacerbated innate and adaptive immune response, and an increased pro-inflammatory state [120]. MYD88^−/-^ (MYD88 adaptor protein) mice present increased lethality and higher brain viral loads related to a dysregulation of cell migration through cytokine modulation [121]. IRF7^−/-^ (IFN regulatory factor-7) mice also develop augmented peripheral and CNS viral burdens [122], and RIG-I^−/-^, MDA5^−/-^, and double knockout (RIG-I^−/-^ x MDA5^−/-^) mice display a complete absence of innate responses and severe pathogenesis [123].

The role of TLRs in flavivirus infection is somehow controversial. For instance, in TLR-7^−/-^ mice a higher susceptibility upon intraperitoneal inoculation was reported, but lack of changes to WNV vulnerability after inoculation by the intradermal route, or by the bite of infected mosquitoes, has also been described in them [101]. TLR-3^−/-^ mice have shown both an increased resistance, suggesting that TLR-3 mediates viral entry to the brain by a transient increase in BBB permeability due to the increment of systemic TNF-α [124], or, by contrast, higher mortality associated with increased viral replication in neurons [125].

PRRs signaling also results in IFN-stimulated genes (ISG) expression with potent and direct antiviral effects in the infected host cell. Both PKR^−/-^ (dsRNA-dependent protein kinase) and RNase L^−/-^ (endoribonuclease RNase-L) mice show increased WNV replication and early viral entry into the CNS [101].

However, it should be noted that the virus has evolved mechanisms of evasion [126]. For example, WNV E protein blocks the production of pro-inflammatory cytokines induced by dsRNA in murine macrophages (Mϕ) by inhibiting the ubiquitination of the receptor-interacting protein 1 (RIP-1) and the subsequent activation of the NF-κB transcription factor [127]. WNV NS proteins block the phosphorylation of the signal transducer and activator of transcription factors STAT1 and STAT2, inhibiting the canonical IFN-I and II signaling pathways [128]. NS1 inhibits TLR-3 signaling probably by interfering with IRF-3 and NF-κB translocation [101] and also antagonizes with IFN-β production, likely by suppressing RIG-I activation [129].

#### Natural killer (NK), γδ T and NKT cells

NK cells are innate lymphocytes that bridge the gap between innate and adaptive immunity, are able to exert direct cytotoxic activities in infected cells, and produce cytokines with important roles in both innate and adaptive antiviral responses. The WNV E protein binds to the NK-activator receptor NKp-44, triggering NK cell activation [130], and antiviral activity of human-derived NK cells has been reported *in vitro* [131,132]; however, its absence in murine models showed no effect on WNV susceptibility [133]. Interestingly, NK cells from patients with previous symptomatic WNV infection present a higher frequency and a more robust response with increased IFN-γ production and skewed NK populations [132].

γδ T cells are a subset of CD3+ T innate cells that recognize antigens of different nature, including common-pathogen patterns, directly or in the context of non-classical MHC class Ib or CD1 MHC-like molecules. TCRδ^−/-^ mice show increased viremia and viral dissemination to the CNS related to an absence of γδ T-derived IFN-γ [134], but certain γδ T-cell subsets have also been related to potential WNV pathogenesis [135]. Its role in linking innate and adaptive immune responses during WNV infection is supported by the fact that activated γδ T cells promote DCs maturation and CD4+ T-cell priming [136], and because TCRδ^−/-^ mice re-exposed to WNV have reduced memory CD8+ T-cell subsets [137].

NKT cells are also a subset of CD3+ T cells that express conventional α/β TCRs of limited variability that recognize lipid antigens presented in the context of CD1d MHC-like molecules [138]. However, to our knowledge, only one study has reported that WNV infection interferes with NKT-DC activity, promoting NKT-derived anti-inflammatory cytokine production [139].

#### The mononuclear phagocyte system (MPS)

MPS is a complex family of cells that share common ancestors, functions, and markers, including monocytes (Mo), Mϕ, and Mo-derived DC (mo-DC), with different roles during WNV infection; so that, human blood-derived Mo and Mϕ sustain WNV infection *in vitro* [140]. In fact, and although increased mortality in CCR2^−/-^ mice was reported [141], later on, the abrogation of CCL-2-dependent recruitment of CCR2+ Mo was associated with an increase in mice survival upon viral challenge [142].

Mϕ are no-terminally differentiated innate cells able to sense pathogens through PRRs present on their surface and to trap and phagocyte pathogens and virus-infected cells that undergo apoptosis. Although a pathogenic role of Mϕ during WNV infection by antibody-dependent enhancement (ADE) was suggested [143], no evidence of its occurrence in natural infections has been observed [144]. WNV infection of primary human Mϕ suppresses the production of pro-inflammatory cytokines, such as IL-1β and IFN-β, thus attenuating its antiviral activity [145]. A protective role of Mϕ during WNV infection has been shown in mice depleted of them, in which increased susceptibility [146] and viral dissemination to the CNS [147] were observed. However, a higher risk of WNV-associated neuropathology in older people due to Mϕ-altered innate responses [148] and a possible role of Mϕ in the “Trojan horse” phenomenon has also been suggested [149].

#### DCs

DCs are considered the most important APCs able to efficiently prime CD4+ and CD8+ T cells. Upon WNV infection, infected DCs, such as LCs, can migrate to SLO, spreading the virus and allowing a second round of viral replication. However, in SLO, infected DCs are also important sources of viral antigen for resident DCs, and can prime T cells to start specific adaptive immune responses that normally clear the virus. In WNV infection, it has been reported that in human-derived DC IFN-α production was dependent on viral replication [150] and that human-derived DCs are not properly activated, which has been proposed to be behind the dysfunctional T-cell responses observed in certain patients which develop neuropathology [151]. Additionally, DCs from old donors have impaired IFN-I responses upon *in vitro* WNV infection, which could be related to the enhanced pathogenicity observed in the elderly [115]. Moreover, blocking DC-IFN-I signaling in mice was reported to have a detrimental effect, leading to virus-induced sepsis [152].

### Adaptive control of the virus

#### Humoral immune system

The humoral adaptive immune response exerted by B cells during WNV infection results in the production of specific neutralizing IgM and IgG Abs that are essential for viral clearance. Early studies performed in µMT and BAFFR-deficient mice devoid of functional B cells, and therefore of Abs, showed an increased susceptibility to WNV infection that was avoided by passive transfer of Abs from immunized mice [153]. Total IgM and IgA seroconversion occurred from day 3 to 9 post-infection, and IgG appears as early as 4 days post-infection. WNV infection in mice elicits Abs specific for the E, prM, NS1, NS3, and NS5 proteins, being only neutralizing those specific for the E and the NS1. Abs raised against the different domains of the E protein have different neutralizing activity, with DIII eliciting the most neutralizing ones. Activation of the complement system is also necessary for protective responses against WNV, and impacts in B cell responses during infection. Hence, mice lacking complement receptors (CRs) show increased susceptibility to the virus with higher viral burden in the CNS and lower levels of both IgM and IgG [154].

#### Cellular immune system

The cellular adaptive immune response is mediated by T cells (CD4+ T helper cells, Th, and CD8+ T cytotoxic cells, Tc) that differ in surface molecule expression, antigen recognition, and effector functions. Both CD4+ and CD8+ T cells are essential for WNV control. Thus, mice deficient for CD8+ T cells show increased susceptibility to the infection, and passive transfer of both naïve and WNV-cognate CD8+ T cells has a positive impact on virus control [155,156].

Naïve Th cells are activated by antigen encounter and depending on the stimuli, like strength or surrounding cytokines, differentiate to several subtypes with different related effector functions, including those of T-regulatory and memory phenotypes important for recall responses. Activated Th1 cells secrete pro-inflammatory cytokines (IL-2) with key roles in Tc activation and in promoting inflammation and immune responses against viruses (IFN-γ).

Mice with genetic or acquired deficiency of CD4+ T cells develop a prolonged infection of the virus in the CNS [157] and, although levels of IgM are not impacted, a decrease of IgG or impaired CD8+ T-cell trafficking are observed, supporting the important role of this subset of cells during WNV infection [157]. CD4+ T cells can also exert cytotoxic activity upon viral infections inducing apoptosis in infected cells. RAG^−/-^ mice devoid of B and T-cell development show high susceptibility to WNV infection, and adoptive transfer of WNV-specific CD4+ T cells significantly protect them, which was linked to a cytolytic activity that was dependent on the Fas/FasL and perforin pathways [158]. In fact, gld mice deficient in FasL expression present high susceptibility to WNV infection, without affecting CD8+ T-cell priming in the periphery, but promoting an increase of viral burden in the CNS and a delayed viral clearance. Even more, the adoptive transfer of wt, but not gld-derived CD8+ WNV-cognate T cells, limits the viral infection, suggesting an important role of CD8+ T cells in killing infected neurons in a FasL-dependent manner [159]. Similar results have been obtained with transgenic mice deficient in perforin [133].

IL-17A is secreted by Th17 cells and is implicated in CNS inflammation by contributing to BBB permeability. It has been demonstrated that IL-17A is positively involved in WNV clearance by promoting CD8+ T-cell cytotoxicity because its deficiency increases mice susceptibility and brain viral burden [160]. Another effector mechanism described for CD8+ T cells in the control of WNV infection was dependent on tumor necrosis factor-related apoptosis-inducing ligand (TRAIL), as TRAIL^−/-^ mice are more susceptible to WNV infection [161]. Moreover, CD40 is involved in T-cell priming, and, consequently, CD40^−/-^ mice exhibit high vulnerability to WNV [162]. On the other hand, CXCL10, CCR5, and CCR7 molecules are related to leukocyte trafficking and impact on T-cell activities during WNV infection. Thereby, mice genetically or antibody-mediated, devoid of CXCL10, CCR5, or CCR7 expression, show increased susceptibility [163–165].

Overall, these data indicate that CD4+ and CD8+ T cells impact the CNS promoting viral clearance, while defective functions in these immune cell compartments do not seem to severely affect viral kinetics in the periphery, even though an impact on humoral responses has been observed.

Although the role of T cells in the clearance of the WNV has been solidly demonstrated, T cells have also been related to immune-mediated pathological effects, as the above-mentioned “Trojan horse” phenomenon. Thus, it has been suggested that both CD4+ and CD8+ T cells are able to transport WNV to the CNS in a Drak2 (death-associated protein kinase-related apoptosis-inducing kinase-2 that is specifically expressed in B and T cells) dependent manner. So that, Drak2^−/-^ mice show a more resistant phenotype to WNV infection characterized by a higher amount of IFN-γ-producing T-cells in the spleen, and reduced viral load and CD4+ and CD8+ T-cell infiltrates in the brain [166].

The different outcomes in humans upon WNV infections have been related, among others, to immune-mediated mechanisms and impairment of immune players that, in turn, are linked to genetic and age-related host risk factors. In this context, old mice show decreased numbers of total Th and B cells [167] and, in a mouse model of age-related susceptibility for WNV, a defective T-cell response in terms of reduced cytolytic activity, cytokine secretion, and multifunctionality has been described. Even more, adoptive transfer of T cells from adult mice, but not from old mice, was able to protect immunodeficient mice against WNV, and a lower infiltration to the brain of CD8+ T cells transferred from old mice was also observed [168].

In humans, the phenotype of CD8+ T cells has been analyzed in patients with diverse clinical outcomes. Increased numbers of WNV-specific T cells were observed in subjects with neuroinvasion and in older subjects, and the CD4+ T-cell repertoire was polarized to restricted phenotypes, with a reduced frequency of T regulatory cells (T regs) in symptomatic donors in comparison with asymptomatic ones [169]. Accordingly, T reg-deficient mice display higher rates of lethality upon WNV infection than wt animals [170].

## Clinical manifestations and pathogenesis

### Humans

WNV infections are asymptomatic in about 80% of people and, when symptomatic, most patients present a mild febrile disease known as West Nile fever (WNF) (https://www.cdc.gov/westnile/symptoms/index.html). Apart from fever, the mild disease is manifested by several nonspecific flu-like symptoms (headache, fatigue, myalgia, arthralgia, weakness, rash, and gastrointestinal problems, including nausea and vomiting that can lead to dehydration) [171]. Less than 1% of infected people develop severe West Nile virus neuroinvasive disease (WND), and symptoms usually appear between 2 to 15 days after infection and last for up to 5 days [172]. WND can be characterized by multiple syndromes, as West Nile meningitis (WNM), West Nile encephalitis (WNE), and West Nile poliomyelitis (WNP) [173]. Genetic factors, underlying diseases (cancer, diabetes, hypertension, renal disease, or transplanted people), gender, age, and immune status (older and immunosuppressed people are more prone to develop severe forms of WND), contribute to virus susceptibility and disease severity [174]. Patients with WNE usually present movement disorders, including severe tremors and Parkinsonism [175]. In fact, WNV is currently considered one of the most important causative agents of human viral encephalitis worldwide [149]. WNP produces a poliomyelitis-like acute flaccid paralysis, which at its most severe presentation can cause quadriplegia and respiratory failure with an estimated 10% of the neuroinvasive cases resulting fatal [173]. Recovery from severe illness might take several weeks or months, and more than 50% of survivors that presented severe symptoms reported physical and cognitive sequelae up to 2 years later [176].

Non-neurological clinical manifestations are less frequent. Ocular pathology has been described upon WNV infection, mainly bilateral multifocal chorioretinitis [177], but also vitritis, optic neuritis, retinal hemorrhage, and iridocyclitis [178]. Likewise, hepatitis, pancreatitis, orchitis, myositis, or myocarditis are considered infrequent manifestations of WNV infection [171,173].

Histopathologic findings in patients with WND include changes in deep nuclei of the brain and anterior horns of the spinal cord, with neuronophagia, perivascular inflammation, microglial nodules and neuronal necrosis in the gray matter, with infiltrates of microglia, polymorphonuclear leukocytes, loss of neurons, and, in few patients, endoneurial mononuclear inflammation of cranial nerve roots and spinal nerves [175]. In patients presenting long-lasting clinical manifestations, focal demyelination, gliosis, and occasional perivascular infiltrates have been observed [179].

Other histopathological findings in non-neurological tissues are scattered hepatocyte necrosis with neutrophilic infiltrates, microvesicular steatosis, and erythrophagocytosis by Kupffer cells in the liver, ophthalmoscopic lesions (focal retinal vascular sheathing and leakage, optic disc swelling, and superior intraretinal hemorrhages), intra-alveolar hemorrhage and edema in the lungs; fibrin thrombi in the small vessels in spleen, lung, or kidney, and scattered inflammatory cell infiltrates in the adrenal glands [178,180].

### Horses

Even though most equine infections remain asymptomatic, approximately 20% of infected horses develop clinical signs, showing a more severe disease than humans [181]. The most common WNV-associated signs, apart from fever, are related to nervous system infection and inflammation. Infected horses can develop ataxia combined with circling, weakness in hind and forelimbs, quadriplegia, paresis, convulsions, hepatitis, chewing, tongue paralysis, pupil miosis, partial blindness, depression, and recumbence [173]. Age and gender also play a role in horse infection, mares are less affected, and, contrary to human, older horses are less likely to become infected. Around 10–20% of horses that recover from neurological disease present residual neurologic deficits [181]. Histopathological findings in horses with affected spinal cords are similar to those of humans with polioencephalomyelitis.

### Birds

Birds are the main vertebrate hosts of WNV and they usually do not present clinical signs, but if so, the most common ones are ruffled feathers, lethargy, difficulty of movements, and loss of appetite that lead to a marked loss of body weight [182,183]. Less frequent signs are abundant oral and nasal secretion, dehydration, reduced fecal output, intermittent head jerking, and convulsions [182]. Mortality in infected birds usually happens within the first 24 h after the presentation of clinical signs [184].

The most observed lesion is necrosis and the most affected organs are the brain (hemorrhages), liver (hemorrhages), heart (myocarditis, myocytolysis, and fibrosis), kidney (nephritis), and spleen (splenomegaly) [182,183], as well as ocular lesions (neuritis, retinal inflammation, and iris degeneration) that can drive to blindness [185]. Other organs are less frequently affected [173].

### Other vertebrates

As commented before, besides humans, horses, and birds, WNV can experimentally or naturally infect a wide variety of wild and domestic vertebrate species. These animals are usually asymptomatic, being the most common signs fever, lethargy, weakness, and tremors, although many others have also been observed sporadically. Similarly, a huge number of histopathological lesions have been documented (Table 1). However, none of these animals seem to play a relevant role in the transmission of WNV in nature [1].

**Table 1.** Common signs in WNV infected vertebrate species

Signs	Vertebrate species	Reference
**Salivation**	Birds; Non-human primates	[182,266]
**Myopathy**	Canids	[267,268]
**Skin lesions**	Crocodiles and alligators	[269]
**Diarrhea**	Seals; Deers; Canids	[268,270,271]
**Vomiting**	Seals	[270]
**Staggering**	Sheep	[272]
**Circling**	Horses; Sheep; Squirrels	[272,273]
**Ocular signs**	Horses; Sheep; Non-human primates; Squirrels; Canids	[266,268,272,274,275]
**Head tilt**	Horses; Birds; Deers; Squirrels; Canids	[182,268,271,274,276]
**Anorexia**	Horses; Birds; Alpacas; Sheep; Seals; Bears; Canids	[182,268,270,272,274,277–279]
**Polydipsia, dehydration**	Birds; Bears; Canids	[182,268,279]
**Tremors, Convulsions**	Horses; Birds; Alpacas; Sheep; Seals; Non-human primates; Deers; Bears; Squirrels	[182,266,270,272,276–281]
**Ataxia**	Horses; Birds; Alpacas; Non-human primates; Deers; Squirrels; Canids	[99,182,183,266,268,273,274,277,278,280–282]
**Fever**	Horses; Alpacas; Sheep; Deers; Canids	[268,271,272,274,277,278]
**Agitation**	Alpacas	[278,282]
**Recumbency**	Horses; Alpacas; Deers	[271,278,280,282,283]
**Ruffled feathers**	Birds	[182,183]

### Persistent infections

WNV persistent infection in humans is difficult to assess [186], however, the presence of the virus in human urine lasting up to 9 years after the infection has been reported, revealing that renal infection can remain for years [187], as well as its presence in brain tissue 4 months after initial diagnosis [188]. Furthermore, persistent IgM has been detected up to 8 years after the onset of symptoms [189]. Hence, WNV might establish a chronic disease in humans.

Viral persistence has been addressed in cell cultures [190,191] and animal models. WNV was recovered from several tissues of experimentally infected rhesus monkeys up to 5 months after infection [192], from hamsters and mice for up to 6 months after infection [193–195], and from naturally or experimentally infected birds up to 9 months, which points to a role in viral overwintering [196].

## Viral pathogenesis

Animal model studies have provided a vast understanding of the pathogenesis of WNV infection. As described in detail above, after primary inoculation, WNV replicates at the epidermal site in keratinocytes, where the virus is amplified and disseminated by the blood stream to different organs, although DCs and neutrophils are also WNV cell targets. Several routes are considered to be involved in the entry of the WNV to the brain [91,149,195,197] i) direct entry by alteration of the BBB permeability, associated to host proteins such as intercellular adhesion molecule (ICAM-1), Drak2, macrophage migration inhibitory factor (MIP), and matrix metalloproteinase 9 (MMP-9); ii) infection of peripheral nerves and olfactory neurons and spread to the olfactory bulb; iii) breaching of the blood–cerebrospinal fluid (CSF) barrier by a “Trojan horse” mechanism mediated by infected immune cells that traffic to the CNS; and iv) entry through axonal transport. Once the virus enters the brain, viral infection can result in neuronal degeneration, but the CNS can be also damaged by the immune response elicited against the pathogen [198].

Different reports have evidenced the induction of apoptosis in neurons [9,32,199,200]. Expression of alpha-synuclein, a neuronal protein directly related to the pathogenesis of Parkinson’s disease and damage to the human brain, has been reported to inhibit viral infection and disease in the CNS [201]. Early data regarding the up or down-regulation of autophagy, another type of programmed cell death, upon WNV infection were contradictory [202,203], but later on it has been demonstrated that single amino acid substitutions in WNV NS proteins can alter the ability of the virus to induce an autophagic response [19,204]. WNV also induces the upregulation of ER stress genes in neurons, thus activating the unfolded protein response (UPR), which can provoke cell death after severe or prolonged ER stress. In fact, WNV triggers the three pathways that modulate the UPR, that is, protein kinase R (PKR)-like endoplasmic reticulum kinase (PERK), inositol-requiring enzyme 1 (IRE1α), and activating transcription factor 6 (ATF6) [19,30,31].

## Diagnosis

Diagnosis of WNV infection is based on the detection of Abs, antigens, RNA, and/or infectious virus. The identification of IgM is usually indicative of active infection, while IgG indicates past exposition to the virus. Detection of antigens, RNA, or infectious virus also indicates active infection; however, this is hampered by the short and frequently low viremia found in the plasma in mammals.

### Serological tests

WNV-specific Abs are detected by IgM capture enzyme-linked immunoassays (MAC-ELISA), indirect IgG ELISA, immunofluorescence assay (IFA), hemagglutination inhibition test (HIT), virus neutralization test (VNT), and plaque reduction neutralization test (PRNT) [205]. For some of these approaches, commercial kits are currently available.

IgM generally appears one week after exposure to the virus and can be present in humans for up to 3 months; however, in some cases, it has been detected up to 500 days after onset, and thus caution should be taken when interpreting the results [189]. An active WNV infection should be confirmed by detecting a 4-fold increase in antibody titers between the acute and convalescent stages of infection [205].

WNV-specific IgG is generally detected shortly after IgM and persists for many years; therefore, the presence of IgG alone only evidences a previous infection. Even though most commercial and *in-house* WNV serology assays are based on structural antigens due to their higher exposition to the host immune system, the NS5 and NS1 can also be valuable alternatives [205].

HIT had been historically used, but now it has been replaced by other tests with greater sensitivity and specificity, like the IFA, that can differentiate between IgM and IgG. In any case, VNT and PRNT, considered the gold standard in its most stringent measurement, PRNT_90_ (90% reduction in the number of plaques), should be used whenever possible. These assays can differentiate between different flavivirus infections when neutralizing titers are ≥4-fold for one virus over the others, and thus, they provide confirmatory assays for the specific diagnosis of WNV infection; however, the technique is complex, time-consuming, and laborious, requires the use of viable virus and cell culture under BSL-3 laboratory conditions, and skilled personnel.

### Virus detection

The use of cell culture methods to isolate infectious virus from serum, CSF, or tissue specimens collected early in the course of illness is feasible using different widely available susceptible cell lines [206]; however, it is not routinely performed due to the typically short duration and low viremia titers recorded in mammals, the time needed to perform it, and the need of skilled personnel and BSL-3 facilities.

### Nucleic acid tests

Molecular techniques are commonly used to detect the presence of WNV RNA in serum, CSF, or tissues. Nucleic acid testing involves a preliminary step of RNA extraction from the samples before amplification of WNV RNA, which may be detectable for an average of up to 4 days before the detection of IgM, and is considered more sensitive than virus isolation. It is commonly used in the context of screening of blood and organ donors.

Standard RT-PCR, nested RT-PCR, and quantitative RT-PCR (qRT-PCR) targeting E, NS1, and/or 3ʹUTR genes have been set up for a variety of samples [207], being the latter the preferred one, as it is the most sensitive. One step further is the use of next-generation sequencing (NGS) that shows even greater sensitivity and allows metagenomic deep sequencing (MDS) [208], although its use in routine diagnosis is far from worldwide implementation, as it is expensive, and needs sophisticated equipment and highly skilled personnel.

## Prophylaxis

### Antiviral compounds

There is no specific drug or therapy licensed for the treatment of WNV, but antiviral discovery against the virus is an extensive field in development and continuous revision [209,210]. Searching for antivirals focuses on two different approaches with unique advantages and limitations: compounds targeting viral components, or direct-acting antivirals (DAAs), and drugs interfering with host factors necessary for viral infection, or host-directed antivirals (HDAs). DAAs are supposed to be specific for the virus and less toxic, whereas they offer low barriers to resistance development. On the contrary, HDAs have a potential broad-spectrum when targeted against conserved factors among related flaviviruses, and a higher barrier to resistance development. Antiviral discovery against WNV presents additional challenges because candidates for therapeutic interventions should be able to cross the BBB and penetrate into CNS.

#### Direct-acting antivirals (DAAs)

DAAs can target different steps of the WNV life cycle. For example, those targeting entry and fusion mechanism mediated by the E protein [211], as arbidol, a broad-spectrum antiviral compound approved in Russia and China for influenza whose mechanism of action is presumably based on inhibition of viral fusion, that impairs WNV infection in cultured cells [212]. Other compounds like polyphenols, monoterpene alcohols, or labyrinthopeptins can bind the viral particle and exhibit virucidal effect [213–215].

Regarding NS proteins, the most prominent WNV targets are the protease and helicase enzymatic activities of NS3, although their clinical application still remains far away, and the methyltransferase and RdRp activities of NS5.

In the context of NS3, a number of studies have addressed the potential of various compounds for inhibition of its protease [216] and helicase activities, like ivermectin, a broadly used anti-helminthic drug [217] that also impairs WNV infection by interfering with the nuclear transport importin machinery [218].

Concerning NS5, its methyltransferase activity, responsible for capping the 5ʹ end of the viral genome, is inhibited in cell culture by BG-323, a lead compound of the 2-thioxothiazolidin-4-one family that exhibits potent anticapping activity, but it has not shown antiviral activity in mouse models [219]. On the other hand, many antiviral candidates against the RdRp activity have been explored. Ribavirin showed good antiviral activity in cultured cells [220] but results from animal models and clinical data did not support its efficacy, and, consequently, the Clinical Practice Guidelines by the Infectious Diseases Society of America did not recommend its use for the treatment of WNV [221]. WNV antiviral activity of nucleoside analogs, such as 7-deaza-2ʹ-C-methyladenosine [222] or 7-deaza-2ʹ-C-ethynyladenosine (NITD008) [223] have been reported in animal models, however, NITD008 failed in preclinical toxicity studies in rats and dogs due to its insufficient safety profiles [224]. Galidesivir (BCX4430), an imino-C-nucleoside currently in clinical trials for YF and COVID-19 (NCT03800173), also impairs WNV replication *in vitro* and also reduces the replication of other flaviviruses in mouse models [225]. Oral administration of the modified pyrazine analog Favipiravir (T-705) was protective against a lethal WNV infection in mice [226]. This drug exerts broad-spectrum antiviral activity and has been approved for influenza in Japan [227], making it an interesting antiviral candidate whose mode of action could be related, among others, to lethal mutagenesis [228]. Sofosbuvir, a licensed nucleoside, also exhibited antiviral activity against WNV in cell culture [229], but its efficacy has still to be demonstrated *in vivo*.

Other DAA approaches included antisense technology therapeutic candidates. Arginine rich peptide-conjugated phosphorodiamidate morpholino oligomers, whose sequences are complementary to RNA elements located in the 5′- and 3′-termini of the WNV genome, showed good antiviral potency without apparent cytotoxicity *in vitro* [230]. In this context, the antisense drug candidate AVI-4020 entered two phase I clinical trials, one in patients with possible acute neuroinvasive WN disease (NCT00091845), and another pharmacokinetic study in adult healthy volunteers (NCT00387283) to find out how much and how fast this drug crosses the BBB.

#### Host-directed antivirals (HDAs)

Multiple cellular factors necessary for WNV infection that can be used for antiviral discovery have been identified [209,210]. Among them, we could highlight strategies based on the use of α-glucosidase inhibitors [231], oligosaccharyltransferase (OST) inhibitors [232], furin inhibitors that prevent prM/E cleavage and particle maturation [233], DEAD-box polypeptide 3 (DDX3) inhibitors [234], or autophagy modulators [19]. Inhibitors of cellular kinases, such as PKC [235] and adenosine monophosphate-activated PK (AMPK) [236] also reduce WNV infection in cultured cells. Moreover, due to the high dependence of WNV infection on host lipid metabolism, compounds targeting it, including that of cholesterol, sphingolipids, or fatty acids, reduce WNV infection in cultured cells [237]. So that, treatment with PF-05175157, an inhibitor of acetyl-Coenzyme A carboxylase that is the rate-limiting enzyme of fatty acid synthesis, reduces WNV infection in a mouse model [238], providing the proof of concept of the feasibility of lipid-targeting antiviral strategies. It is also important to consider that several drugs already licensed for clinical practice to treat illnesses other than WN disease could also exhibit some antiviral effect, such as the ivermectin mentioned above [217], mainly if they can cross the BBB. Preclinical studies with antiparkinsonian compounds (L-dopamine, selegiline, isatin, and amantadine) or the antiepileptic valproic acid [239,240] support this approach.

Among HDAs, another complementary approach is based on the exploitation of host restriction factors via immunostimulatory compounds. This is the case of IFN-α, or its inducers, which showed favorable effects in preclinical models [241,242]. In fact, the first WNV treatment trial approved in the US considered the utilization of IFN-α-2b [243]. However, there is still limited evidence of the efficacy of this treatment due to the reduced number of cases analyzed [244], together with reports on treatment failure [245]. Therefore, the efficacy of IFN-α-2b for the treatment of WNV remains to be convincingly demonstrated.

#### Therapeutic Abs

Passive immunization with immune serum or monoclonal Abs for therapeutic or prophylactic use could be useful for the control of WN disease, especially in immunocompromised patients, as evidenced in animal models [246,247]. The most promising therapeutic antibody candidates are those targeting E glycoprotein (Table 2). Clinical applications of passive immunization against WNV started with intravenous immunoglobulin (IVIg) administration that reduced the severity of the disease in some cases when administered early, although its efficacy greatly differed among the sera pools used, probably due to the different content in NAbs [248]. In this context, Omr-IgG-am, an IVIg containing WNV-specific Abs, has completed phase I/II trials [249]. Regarding therapeutic monoclonal Abs, the most advanced candidate was MGAWN1, a humanized monoclonal antibody (IgG1k) targeting DIII that neutralizes WNV and does not react with other flaviviruses [250,251]. Its safety, tolerability, and therapeutic potential was demonstrated in a clinical trial (NCT00515385), showing that the concentration reached in the CSF of treated patients was higher than those required to neutralize the virus in hamster models [252]. However, the subsequent advanced phase II study (NCT00927953) was terminated due to the inability to enroll.

**Table 2.** Clinical trials related to passive immunization strategies against WNV

Biological	Description	Clinical trial identifier (NCT Number)	Phase	Start	Reference	Study purposes
Omr-IgG-am	IVIg containing antibodies specific for WNV	NCT00068055	I/II	2003	NA	To assess whether Omr-IgG-am is safe and well-tolerated in patients with suspected or laboratory-diagnosed WNV disease, and initial estimation of efficacy
		NCT00069316	II	2003	[249]	
MGAWN1	Humanized monoclonal antibody to WNV	NCT00515385	I	2007	[252]	To evaluate the safety, tolerability, and pharmacokinetics of escalating doses of MGAWN1 administered as a single intravenous infusion to healthy adults
		NCT00927953	II	2009	NA	To evaluate the safety, efficacy, and pharmacokinetics of MGAWN1 in subjects with WN fever or a syndrome compatible with WN neuroinvasive disease
		NCT01206504		2010	NA	Expanded Access to MGAWN1 in subjects with suspected WN neuroinvasive disease; suspected WNV infection, or substantial accidental exposure

NA: Not available. ClinicalTrials.gov identifier (NCT number)

### Vaccines

#### Veterinary vaccines

Veterinary vaccines have focused on preventing infection in animals vulnerable to WNV, mostly equids, and some of them have already been approved [253]. Up to six equine vaccines against WNV have been licensed, being currently four of them on the market (Table 3). Licensed vaccines include those following classical approaches based on whole virion inactivated vaccines, such as WN-Innovator® that was the first equine vaccine against WNV, is in use since 2001, and was licensed by the USDA in 2003 [254]. Other approaches were based on the expression of prM and E proteins by recombinant live attenuated viruses (either canarypox or YFV), or even via a DNA plasmid platform, which became the first DNA vaccine licensed by the USDA for use in horses [255], although it was later removed from the market. WNV veterinary vaccines have proved to be protective, and their use greatly contributed to reduce the incidence of WN disease in horses in the US [255,256]. Although most vaccines have been based on American strains from Lin 1, the high degree of cross-reactivity between viruses from Lin 1 and 2 supports the efficacy of veterinary vaccines to prevent outbreaks associated with both lineages [184,257–260]. Notwithstanding, despite the proven efficacy of veterinary vaccines, they still exhibit some limitations, like the requirement for repeated administrations for initial immunization, and the relatively short duration of the immunity that makes annual boosters necessary. Therefore, these aspects must be addressed to improve current vaccines.

**Table 3.** Selected clinical trials of WNV vaccines

Type of vaccine	Vaccine name (Developer)	Immunogen	Clinical trial identifier (NCT Number)	Phase	Start	Reference	Relevant results
Live attenuated	ChimeriVax-WN02 (Sanofi Pasteur)	Chimeric vaccine encoding WNV pM and E genes in the backbone of YFV 17D	NA	I		[284]	Safe, well-tolerated, and induced high levels of neutralizing antibodies and CD4+ and CD8 + T cell responses
			NCT00442169	II	2005	[285]	Highly immunogenic in younger, adults and the elderly, including subjects ≥65 years old
			NCT00746798	II	2008	[286]	Highly immunogenic and well tolerated among subjects ≥50 years old
	rWN/DEN4Δ30 (NIAID)	Chimeric vaccine encoding WNV prM and E genes in the backbone of DENV-4 with a 30 nt deletion	NCT02186626	I	2014	[287,288]	Safe and immunogenic in healthy adults, including those aged 50–65
DNA	VRC-WNVDNA017-00-VP (NIAID)	DNA vaccine encoding the prM and E genes	NCT00106769	I	2005	[289]	Safe and well-tolerated. Induced T cell and antibody responses
	VRC-WNVDNA020-00-VP (NIAID)		NCT00300417	I	2006	[290]	Safe and well-tolerated. Induced T cell and neutralizing antibody responses, similar responses in young and older age group
Subunit	WN-80E HBV 002 (Hawaii Biotech)	Recombinant, truncated E protein	NCT00707642	I	2008	[291]	Safe and induced seroconversion
Inactivated	Formalin-inactivated WNV (Nanotherapeutics Inc.)	Formalin-inactivated whole virus		I/II		[292]	Safe and immunogenic
	HydroVax-001 (Najit Technologies)	Hydrogen peroxide-inactivated whole virion	NCT02337868	I	2015	[293]	Modestly immunogenic and well-tolerated

NA: Not available. *ClinicalTrials.gov (NCT number) identifier is indicated when available*

#### Human vaccines

As mentioned above, no human vaccine has been approved to prevent WNV infection and none has even progressed from phase I/II clinical trials (Table 4). Overall, these vaccines follow similar approaches to those of veterinary vaccines. The immunization schedule has differed among trials, but almost all of them required multiple doses, except those based on live attenuated viruses using either YFV (ChimeriVax) or DENV-4 (rWN/DEN4Δ30) as vectors to express WNV prM and E proteins [253]. In most cases, the assessment of the immunogenicity was based on seroconversion and detection of NAbs, although in some cases, the development of a specific T-cell response was analyzed. Overall, most vaccines were well-tolerated, safe, and immunogenic in both young adults and elderly volunteers, pointing to their feasibility, but no phase III trial assessing the real efficacy and safety of any human vaccine against WNV has been initiated. In addition, the potential for disease enhancement associated with preexisting immunity to heterologous flaviviruses cannot be discarded. In fact, hemorrhagic manifestations of WNV infection in a patient with prior DENV infection history has been documented [180]. Exacerbation of ZIKV infection by preexisting WNV immunity has been proposed in mouse models [261], and the presence of sub-neutralizing antibody concentrations has been suggested to be a risk factor for ADE of WNV infection [262]. Nevertheless, other reports support a certain degree of cross-protective immunity between WNV and other flaviviruses [263,264]. Therefore, detailed studies to analyze the potential effects of cross-reactivity between WNV and co-circulating flaviviruses should be performed to warrant the safety of future human vaccines against WNV.

**Table 4.** Equine vaccines licensed against WNV

Type of vaccine	Vaccine name	Immunogen	Status
Inactivated	West Nile Innovator (US) Equip WNV (Europe)	Inactivated whole virus	In the market
	Vetera WNV	Inactivated whole virus	In the market
	Prestige WNV	Whole inactivated WNV – flavivirus chimera	In the market
Live attenuated virus	Recombiteq Equine WNV (US) Proteq West Nile (Europe)	Chimeric vaccine consisting in a canarypox expressing WNV prM and E	In the market
	PreveNile	Chimeric vaccine encoding WNV pM and E genes in the backbone of YFV 17D	Recalled
DNA	West Nile Innovator DNA	Plasmid encoding prM and E	Discontinued

### Preventive measures

Prevention plays a very important role to reduce the impact of WNV and, by now, the most efficient strategy to avoid infection is to elude mosquito bites (https://www.cdc.gov/westnile/prevention/index.html). Simple control measures, such as the use of suitable mosquito repellents and the reduction of the exposed skin surface by wearing long sleeves and long pants can reduce the risk of mosquito bites. Many mosquito species have peaks of activity during dusk and dawn, and, thus, reducing outdoor activity at these times contributes to reduce the risk, as well as the installation of screens on doors and windows. Other easy control measures that can be locally implemented are those aimed to prevent mosquitoes from laying eggs in or near stagnant water, such as minimizing items that hold water (tires, buckets, planters, toys, pools, birdbaths, flowerpots, or trash containers). These preventive measures together with the support of mosquito control programs and community-based WNV control actions will also contribute to reduce the risk of the disease via a more efficient surveillance.

## Conclusions

Globalization and climate change are driving the spread of zoonotic viruses, as exemplified by the recent epidemics/pandemics of avian influenza, Ebola, Zika, SARS, MERS, and, the most recent and devastating SARS-CoV-2. These facts have highlighted the need to implement control measures before the risks become uncontrollable and, therefore, a deep understanding of the characteristics and behavior of the pathogens and their interactions with the environment, animals, and humans is necessary.

Among the most relevant (re)emerging arboviruses are mosquito-transmitted flaviviruses, such as Dengue, Zika, and West Nile viruses, which account for millions of infections and thousands of deaths among humans, mammals, and birds worldwide. Since the beginning of this century, WNV (re)emerged in different geographic areas around the world with an increase in the number, frequency, and severity of outbreaks. Although since its first description, more than 80 years ago, our knowledge about WNV infection has greatly increased, some aspects still need to be further addressed. Among them, the role that climate (temperature, humidity, etc.) and anthropogenic factors play in WNV spread; the reasons for the different clinical manifestations observed around the world and its long-term sequelae; a deep understanding of WNV pathogenicity, virulence, and immunity; the implementation of surveillance programs and better diagnostic tools; and the search for antivirals and cost-effective human vaccines. Advances on our current knowledge on WNV infection will greatly help to fight not only its future expansion to new niches around the world, but also of other emerging RNA viruses, and more precisely of other arboviruses. To achieve these goals is necessary that the national and international authorities will be aware of the risks and take the necessary joined measures to implement infrastructures, training, tools, methodologies, and funding.
